# Excrescence on the Eye-ball—Removal—Recovery

**Published:** 1847-04

**Authors:** J. Evans

**Affiliations:** Prof. of Obs., &c., in Rush Medical College


ARTICLE III.

*Excrescence on the Eye-Ball — Removal — Recovery:* By J. Evans, M. D., Prof. of Obs., &c., in Rush Medical College.

In the fall of 1839, Mr. E., of middle age, a farmer, of sanguine temperament and robust constitution, consulted me in reference to a tumor on the globe of the eye, which had made its appearance in the form of a small pearly spot some two years previously, and had gradually increased in size, notwithstanding numerous washes and escharotic applications, up to this time. It originated upon the sclerotic coat, near its junction with the lucid cornea, in the inner canthus, and extended, in its growth upon the cornea, so far as to be directly over the margin of the pupil when moderately dilated. It was of the size and shape of two-thirds of a pea, white and opaque, though not of the pearly whiteness of the albugenia. It obstructed vision in the eye by discoloring a portion of the cornea, and interfered with the palpebræ, causing irritation occasionally from the friction produced by their closing over it, which were all the inconveniences caused by it.

On the fourth of September I excised it; but fearing to cut deep lest I should injure the coats of the eye, the whole of the diseased mass was not removed. It was but a short time until it was as large as ever, notwithstanding it was frequently touched with nitrate of silver.

*September* 18. Second operation. I now passed the knife through so as to follow a right line from the base of the tumor on one side to the corresponding point opposite to it, leaving a flat surface upon the globe the size of the base of the excresence. But, in a few days, a growth appeared in the centre of this space, of the same character and shape of the original one, which attained to about half its size.

*October* 13. Third operation. This was performed in the same manner as before. Having a much smaller base, I ventured to cut deeper and removed all the diseased structure. There was but little inflammation excited, the patient being subjected to a strict antiphlogistic regimen after each operation.

I did not see the patient for some months, when he informed me that a pimple the size of a pin head had made its appearance, soon after the last operation, upon the disputed territory, but that a *charm* doctor passed his hand over the eye one evening, and nothing had been seen of it since!!!

At first sight this resembled a case of partial staphyloma; but, on minute examination, it was easily distinguished to be a growth upon the surface of the eye.

The substance of the tumor resembled fibro-cartilage.

*January* 1845. Four years after the operations, no sign of a return of the disease. The site of the tumor is marked by a flattened surface, covered with the conjunctiva and healthy, of the size of the base of the original excrescence.

